# Strain Localizations in Notches for a Coarse-Grained Ni-Based Superalloy: Simulations and Experiments

**DOI:** 10.3390/ma14030564

**Published:** 2021-01-25

**Authors:** Francesco Sausto, Luca Patriarca, Stefano Foletti, Stefano Beretta, Erica Vacchieri

**Affiliations:** 1 Department of Mechanical Engineering, School of Industrial and Information Engineering, Politecnico di Milano, Via La Masa 1, 20156 Milano, Italy; francesco.sausto@polimi.it (F.S.); stefano.foletti@polimi.it (S.F.); stefano.beretta@polimi.it (S.B.); 2 Ansaldo Energia, SPA, Via N. Lorenzi 8, 16152 Genova, Italy; erica.vacchieri@ansaldoenergia.com

**Keywords:** coarse-grained Ni-based superalloys, crystal plasticity, notch effect, strain concentration factor

## Abstract

Alloys used for turbine blades have to safely sustain severe thermomechanical loadings during service such as, for example, centrifugal loadings, creep and high temperature gradients. For these applications, cast Ni-based superalloys characterized by a coarse-grained microstructure are widely adopted. This microstructure dictates a strong anisotropic mechanical behaviour and, concurrently, a large scatter in the fatigue properties is observed. In this work, Crystal Plasticity Finite Element (CPFE) simulations and strain measurements performed by means of Digital Image Correlations (DIC) were adopted to study the variability introduced by the coarse-grained microstructure. In particular, the CPFE simulations were calibrated and used to simulate the effect of the grain cluster orientations in proximity to notches, which reproduce the cooling air ducts of the turbine blades. The numerical simulations were experimentally validated by the DIC measurements. This study aims to predict the statistical variability of the strain concentration factors and support component design.

## 1. Introduction

Nowadays, renewable energy plants are increasingly used for energy production, even if they cannot guarantee a continuous supply of electrical energy. Thus, turbo-gas engines are still used as an alternative source of energy able to maintain the continuous production of energy. For this reason, industries are still working on improving the design of gas turbines. In particular, frequent start-ups and shut-downs of these power plants lead to fatigue problems in the engine components, which are particularly burdensome for blades and disks in the high pressure and high temperature turbine stages. One of the materials available on the market which can sustain the high mechanical and thermal loads is the Nickel-based superalloy René80 [1]. To guarantee high creep and fatigue performance at high temperatures, René80 features a coarse-grained microstructure. This microstructure directly influences the mechanical behaviour of this material, leading to high anisotropy in its mechanical properties, which promotes a large scatter in the fatigue life. The usual practice for taking into account the great fatigue variability of this material is the employment of a large safety factor. Due to the pressing requirement to improve the performance of these turbo-gas engines, new design methodologies are required, which properly consider the variability introduced by the coarse microstructure.

The possibility to predict the scatter of the fatigue life of René80 correlated with the coarse-grained microstructure and to adopt a proper probabilistic approach can be achieved by combining advanced simulation tools and a dedicated experimental campaign. In [2], Gottschalk and co-workers computed the Schmid factors grain by grain from electron back-scattered diffraction (EBSD) data of the René80 specimens tested. The Schmid factor depends on the orientation of the grain considered in relation to the principal loading axis, and it is hence a parameter that depends on the material’s microstructure. Gottschalk et al. proposed correlating the material fatigue life with the Schmid factor, by means of a modified version of the Coffin–Manson curve. The experimental points correlate quite well with the proposed curve, and the authors suggested using the statistical distribution of the Schmid factors in correlation with the proposed curve in the design phase in order to estimate the failure probability of the components. 

One of the drawbacks of this approach is that the Schmid factor depends not only upon the grain orientation in relation to the loading axis, but also on the local stress tensor generated between the neighbouring grains. To overcome this limitation, Engel et al. [3] performed several Monte Carlo simulations of the material microstructure of René80; the polycrystalline models of the specimens obtained were then simulated considering a local elastic anisotropic model by means of the finite elements (FE) technique. From the FE simulations of the microstructure, the Schmid factor was then calculated for each node of the elements. The statistical distribution of numerical values was used in a probabilistic framework and showed a better correlation with experimental results than the work by Gottschalk et al. [2]. This model neglects the plastic strain that a grain can accumulate, even if the material behaves elastically at a macroscopic level.

A refined microstructural model, that takes into account both plastic and elastic strain accumulation at a grain level, is the Crystal Plasticity (CP). The backbone of the model’s CP is based on the theory of deformation of the crystals proposed by Asaro [4,5]. CP can be implemented in finite element (FE) software to simulate the local behaviour of the material’s microstructure, which was proved to correctly match the experimental observations [6,7,8,9]. This numerical technique can be used to estimate the impact of geometrical discontinuities, such as notches or defects, on the local stress and strain behaviour of the material. CPFE simulations were used in the work by Battaile et al. [10] to estimate the scatter of the plastic stress inside the microstructure for several defect dimensions. The main result of this work was that a pore influences the mechanical behaviour of the microstructure when its dimensions are comparable with the grains’ diameter, leading to a high level of scatter of the plastic strain accumulation. Following the results of Battaile et al. [10], Prithivirajan and co-workers [11] studied the critical pore size that interacts with the microstructure of Inconel718 produced by additive manufacturing (AM). The authors showed that the microstructure of this material is influenced by pores or defects that are bigger than 20 μm; pores or defects smaller than this dimension produced a stress gradient that is almost the same as if one considers the material as isotropic and homogeneous.

In this paper, we analyse the notch effect on the mechanical behaviour of the Ni-based superalloy René80. The performance of a gas turbine blade can be increased by air cooling by means of air ducts inside its body. These ducts can be seen as notches, and hence preferential locations for fatigue crack nucleation. The dimensions of these features are comparable with the material’s microstructure, producing an even more accentuated life scatter compared to smooth specimens tested in the laboratory. To estimate the scatter due to the interaction between the notches and the microstructure, the distribution of the strain concentration factor $K_{\varepsilon }$ was estimated both experimentally and numerically by means of CPFE simulations. The model’s CP parameters were estimated from tensile tests on micro-tensile specimens integrated with local, grain-scale, strain measurements using the Digital Image Correlations (DIC) technique. The scatter of the strain concentration factor represents the uncertainty due to the effect of the microstructure, and this has to be accounted for along with the material’s fatigue life variability in the design phase to make a safe assessment of the component considered.

This paper is organized as follows. In Section 2, the material and the experimental set-up are presented and discussed. In Section 3, the experimental results are shown. Section 4 describes the procedure adopted to fit the model’s CP parameters. In Section 5, both the numerical and the experimental results are discussed.

## 2. Material and Experiments

### 2.1. Microstructure of René80

**Figure 1:**
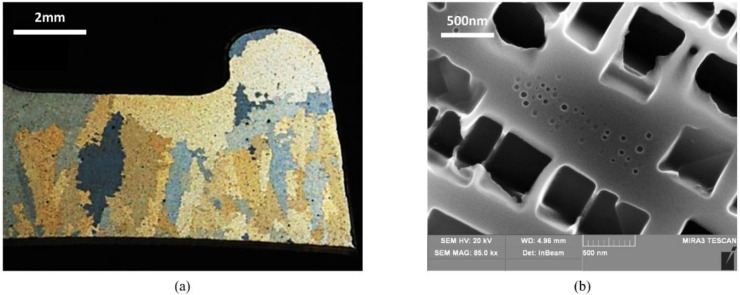
Stereo-microscope micrographs of René80: (**a**) cross section of an actual component; (**b**) γ/γ’ phase microstructure at high magnification in FEG-SEM in the as-delivered condition after metallographic etching to highlight γ’ phase.

René80 is a coarse-grain material and the grain size is observed to be quite inhomogeneous, as depicted in Figure 1a for test bar metallographic sections and for real thin wall airfoil components. The starting microstructure for René80 is composed of a bimodal distribution of γ’ phase; the primary particle size is about 400 nm and secondary particles are smaller than 20 nm, with a total volume fraction that reaches about 50%, as shown in Figure 1b.

### 2.2. Digital Image Correlation Set-Up

**Figure 2:**
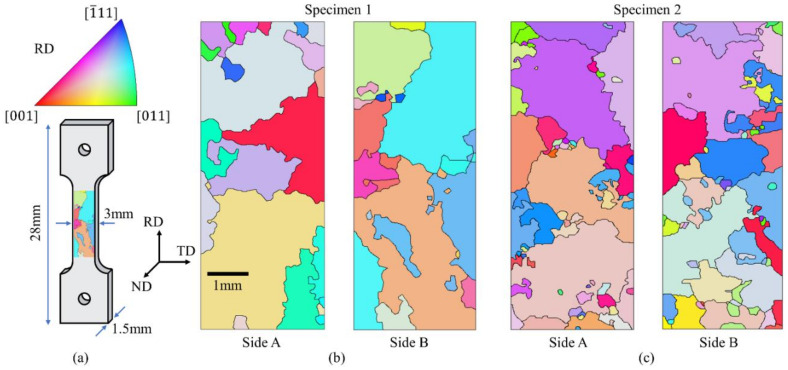
Experimental set-up adopted to calibrate the material’s Crystal Plasticity (CP) parameters: (**a**) the dog-bone tensile specimens; (**b**,**c**) the electron back-scattered diffraction (EBSD) orientation maps obtained on both sides of specimen 1 and specimen 2.

Calibration of the CP parameters was performed based on two dedicated tensile tests. Four specimens were cut by electro-discharge machining (EDM), and their geometry is depicted in Figure 2a. The specimens were initially polished to a surface quality for electron back-scattered diffraction (EBSD) analysis by means of emery papers with a grit size of P800 to P2500, 1 µm diamond paste, and final polishing performed using colloidal silica. Three EBSD maps (approximate dimension 3 mm × 2.5 mm) for each specimen side were acquired by means of an FEG-SEM Mira3 manufactured by Tescan (Brno, Czech Republic) equipped with a Hikari EBSD camera manufactured by EDAX-TSL (Mahwah, NJ 07430, USA). The maps were successively stitched to cover the entire gauge length, and a total of two EBSD maps for each tensile specimen were then obtained. Two tensile specimens were tested, and the stress-strain curves were used to calibrate the material’s CP parameters. The additional EBSD maps measured on the other two specimens were only used to consolidate the CP simulations and define the scatter in the material behaviour induced by the coarse-grained microstructure. The EBSD maps of the two specimens specifically used for CP calibration are shown in Figure 2b,c. The orientation maps are viewed from the two sample sides to properly observe the grain morphology’s development throughout the specimen’s thickness. Despite most of the grain sizes being larger than the specimen’s thickness, the majority of the grains detected on one side of the specimen differ from the grains observed on the opposite side. Calibration of the material’s CP parameters was then performed by analysing both of the EBSD maps for each specimen.

The DIC measurements were performed according to two strategies which, in general, depend on the resolution and dimension of the region of interest (ROI) required. The ex-situ DIC measurements refer to strain measurements performed before and after specimen deformation. Providing a fixed image resolution, the ROI can also be enlarged since multiple images can be acquired and then stitched. The strain maps obtained refer to the un-loaded configuration (residual strains). The second acquisition strategy is labelled as in-situ, and it enables us to measure real time strain fields. The ROI is fixed and the images are continuously acquired during the specimen deformation.

Following the EBSD measurements, the tensile specimens were successively prepared firstly for DIC measurements. A central area with dimensions of 7 mm × 3 mm was marked with a tape and successively etched with a solution of 95 vol.% of hydrochloric acid and 5 vol.% of hydrogen peroxide for 12 s. This region corresponds with the EBSD map acquired previously. A set of optical images of the grains was then captured and stitched. The reconstructed grain geometry was used to properly correlate the DIC measurements with the microstructure. In fact, the EBSD measurements introduce some degree of distortion when the scanned area is large, as in this analysis. To avoid this problem, the geometry was reconstructed using an optical microscope, while the associated grain orientation was extracted from the EBSD data file.

**Figure 3:**
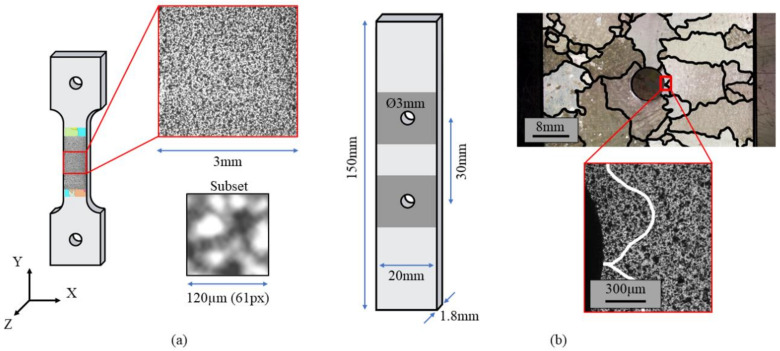
Details of the speckle pattern adopted for the Digital Image Correlations (DIC) measurements performed on the specimens tested: (**a**) speckle pattern of the tensile micro specimen with details of the monitored DIC in-situ region; (**b**) details of the notched specimen with details of the microstructure surrounding the manufactured hole and the speckle pattern adopted to perform the DIC.

The specimen surfaces were painted with a black paint using an IWATA airbrush with a characteristic nozzle diameter of 0.18 mm. The images for the DIC measurements were captured with a final resolution of approximately 2 µm/px, and the ROI covered by the single image was found to be 3.2 mm × 2.4 mm as indicated in Figure 3. The DIC set-up consists of: (i) a 2 megapixel Allied vision Manta G201B CDD digital camera; (ii) a set of lenses manufactured by Optem; (iii) a set of linear micro-stages; (iv) a Schott ACE I EKE rim light fibre optic illuminator. DIC measurements were then performed ex-situ and in-situ. With ex-situ, the area of investigation can be larger than the single ROI as multiple images can be captured to cover the entire speckled surface. Three images were captured along the loading direction before specimen loading, and we captured another three images of the same regions after specimen deformation, at zero load. The correlation of the images made it possible to calculate the strain maps of the entire gauge length of the specimen and correlate this with the EBSD data acquired previously. The strain maps acquired with the ex-situ DIC strategy show the residual strains on the tensile test. The DIC was then used in the in-situ configuration. This set-up requires that the position of the microscope be fixed during the experiment, meanwhile a video is recorded during deformation. The ROI is fixed and the strain measurements are performed continuously. This set-up makes it possible to perform real-time acquisition, and the data can be used to calculate either the averaged stress-strain curve or the strain maps.

The tests were performed under a Deben load machine with a load capacity of ±5 kN. The tests were conducted in displacement control with a displacement rate of 1.5 mm/min at room temperature, and the nominal (bulk) axial strain was measured from the in-situ DIC strain fields.

The notched specimens were machined by means of electro discharge machining (EDM). This process leaves a layer of oxides on the machined surface that are not suitable for testing. To eliminate the material layer affected, specimens were mechanically polished by means of emery papers with meshing of the abrasive particles ranging between 800 and 2500. Two holes were machined in the centre of the plate to act as stress-raisers (Figure 3b).

Before testing, the specimens were airbrushed with black paint to obtain a random speckle on the surfaces, see the inset positioned at the bottom right of Figure 3b. The strain gradient due to the effect of the notch is highly influenced by the microstructure due to the comparable dimensions between the radius of the notch and the nominal size of the grains.

A total of four notched specimens were prepared and tested at room temperature. The tests were conducted in force control by employing an MTS 810 servo-hydraulic testing machine with a maximum load cell capacity of 100 kN. The applied axial force was set to obtain a nominal stress of 350 MPa. The test consisted of two main parts. The initial loading was applied incrementally; at each increment the image of the deformed speckle was recorded in order to evaluate the strain map’s evolution around the notches. Then, the specimens were cyclically loaded at stress ratio $R_{\sigma }=0$ and a frequency of 15 Hz, with a maximum stress of 350 MPa. Fatigue tests were performed discontinuously with interruptions every *N_i_* cycles—this represents a fatigue block. The DIC acquisition was performed at the end of each fatigue block, making it possible to both inspect the presence of a crack and to compute the evolution of the plastic strain accumulation during the test. The material behaves elastically at 350 MPa, reaching a local yielding at the root of the notches.

## 3. Experimental Results

### 3.1. Tensile Tests

**Figure 4:**
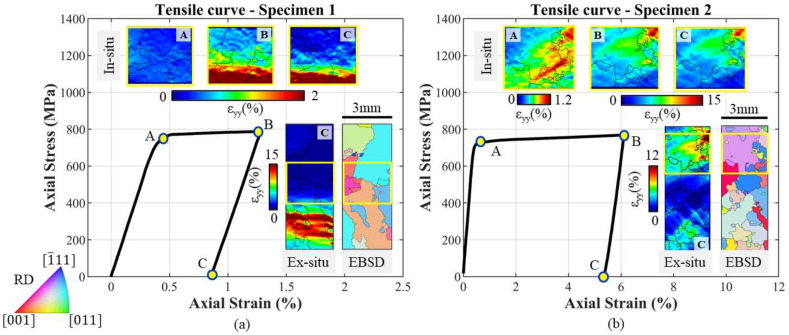
Tensile behaviour and DIC strain measurements for (**a**) Specimen 1 and (**b**) Specimen 2. The strain fields reported in the top were captured in-situ at points A, B and C. The ex-situ DIC strain maps were acquired for the entire specimen region and are reported along with the EBSD data. The yellow box in the ex-situ and in the EBSD map represents the position of the region of interest (ROI) acquired in the in-situ configuration.

Two specimens of the type depicted in Figure 3a were tested in order to calibrate the CP parameters. The tensile curves, obtained from the correlation between the signal from the testing machine’s load cell and the mean strain measured with DIC in the ROI, are reported for specimens 1 and 2 in Figure 4a,b, respectively, by means of the solid black line.

For each test, the strain maps are shown for the ROI related to three points, A B and C, on the tensile curves; all the shown strain maps were computed using a Green-Lagrange tensor. Point A corresponds to the initial yielding phase; at this point most of the grains are subjected to elastic deformation, with sporadic zones in which the plastic strain starts to accumulate. In point B, the unloading phase starts. This point corresponds to the maximum level of macroscopic strain. From the microstructural point of view, the softer grains are subjected to local high yielding as shown in Figure 4. As can be noted, a few numbers of the observed grains, for both tests, show high yielding level, while the rest are subjected to a lower level of deformation. Point C corresponds to the end of the test. At a microstructural level, the residual strain accumulated in the most deformed grains can be observed.

The strain evolution of the ROI was monitored at each time instant of the test. The whole specimen surface was investigated ex-situ for both tests, and this makes it possible to pinpoint the grains with the highest level of accumulated plastic strain. The resulting strain maps corresponding to the ex-situ analyses are reported in Figure 4, together with the experimental tensile curves. On analysing the ex-situ strain map for the first specimen, it can be noted that the position of the ROI is far from the zone of maximum accumulated strains. At the beginning of the first test, only the EBSD map was available, hence it was difficult to estimate a priori the zone of the surface which is more prone to deform. For this reason, the central part of the specimen was monitored in-situ. Differently from the first test, the microstructure of the second specimen was simulated by CPFE with the parameters found in the first test. Thanks to this preliminary simulation, it was possible to monitor the zone with the higher local plastic strain evaluated via FE. As can be noted, the ROI of the second specimen corresponds to the zone of maximum accumulated plastic strain highlighted in the ex-situ map.

The EBSD maps obtained for the two specimens’ observed surfaces are reported together with the strain maps from the ex-situ analysis. In the first test, the most deformed grain featured an orange colour on the EBSD map. In the stereographic triangle (see Figure 2), this colour corresponds to a grain oriented with the [1] crystallographic direction close to the main loading axis. The DIC analysis in Figure 4a confirms that this grain is prone to plastic deformation. In the second test, the violet bigger grain is the one which features the higher level of deformation. Looking at the stereographic triangle of Figure 2, this grain is oriented with the [111] crystallographic direction close to the main loading axis.

### 3.2. Fatigue Tests on Notched Specimens

**Figure 5:**
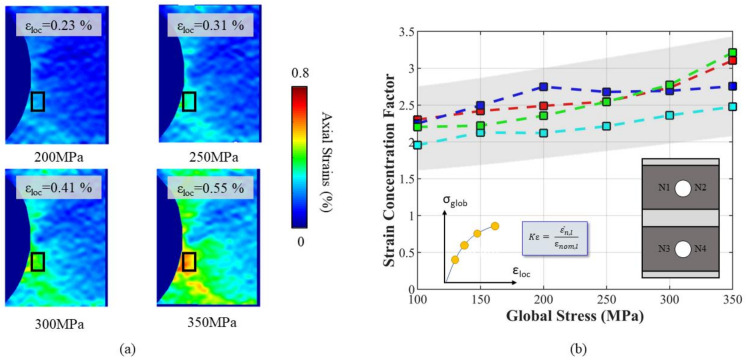
Local strain fields captured during the first loading cycle for the notched specimen. (**a**) The local strains are calculated according the point of local maximum and are averaged in a small rectangular area. (**b**) The local strain data were used to calculate the strain concentration factor $K_{\varepsilon }$ as a function of the applied remote stress σ_glob_ and the local microstructure, which determines the final experimental scatter. Each root of the hole represents a notch, hence for each specimen four notches can be evaluated as shown in the scheme.

The first load reversal was applied incrementally; after each load increment the image of the deformed speckle was acquired. The correlation between these images makes it possible to calculate the evolution of the strain map at different load levels. The load was applied considering an increment of 50 MPa from the initial unloaded state to the maximum deformed state. The resulting strain evolution around the notch of one of the specimens tested is reported in Figure 5a.

The maximum deformation was localized below the centreline of the hole. On increasing the load, one observes that the strain continues to accumulate. Due to the microstructure, the strain concentration is accumulated in the softer grain along the notch’s circumferences, resulting in deformed regions different from the notch root.

At each loading increment, it was then possible to evaluate the evolution of the strain concentration factor from the DIC analysis. The strain concentration factor $K_{\varepsilon }$ at each *i*-th loading increment was computed according to Equation (1):

(1) $$K_{\varepsilon ,i}=\frac{\varepsilon_{i,loc}}{\varepsilon_{i,nom}}$$

where $\varepsilon_{i,nom}$ is the specimen’s nominal strain computed as the ratio of the applied stress and the material’s Young’s modulus, and $\varepsilon_{i,loc}$ is the local maximum strain. The local maximum strain $\varepsilon_{i,loc}$ was computed from DIC strain maps as the average strain inside a control area as shown in Figure 5a. This procedure was necessary due to the intrinsic experimental noise that could affect precise strain calculations from DIC. The size of the control area was set constant for the whole set of experimental measures, with main dimensions of 210 × 310${\;\mu m}^{2}$. The position of the control area was chosen manually for each test in order to precisely localize the maximum zone of deformation.

The local strains obtained from DIC analysis at different applied loads are reported in Figure 5a. In Figure 5b, the experimental strain concentration factor $K_{\varepsilon }$, evaluated at four notch locations, i.e., the four notch roots of the manufactured holes, of one test is reported for different levels of applied load. All the locations feature the same incremental trend, with slight fluctuations due to the unavoidable experimental uncertainty. As can be noted, the values of $K_{\varepsilon }$ show great variability at each load increment from the same specimen. This variability is influenced by the microstructure, which rules the concentration of strain in zones randomly distributed along the notch radius. To appreciate the statistical variability of the experimental findings, a scatter band is depicted in Figure 5b and is shaded in grey, together with the $K_{\varepsilon }$ curves obtained.

**Figure 6:**
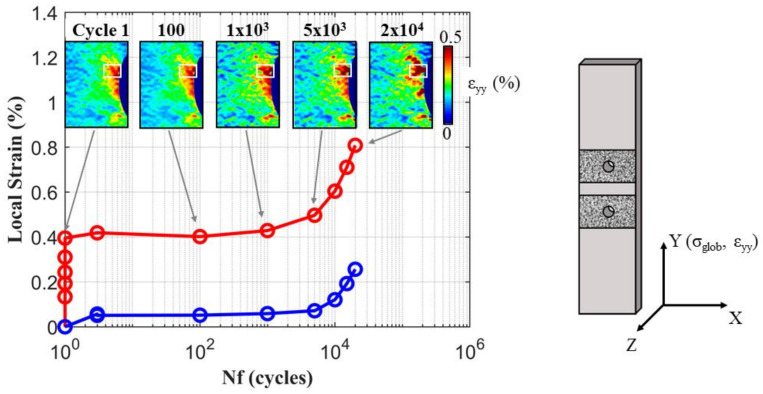
The evolution of the local strains according the number of cycles for a notch showing a fatigue crack. At first, a localization of strains can be observed, which stabilizes in the first cycles. Successively, the rapid increment of local strains indicates the nucleation and propagation of a fatigue crack.

After the first load reversal, the specimens were cyclically tested considering a stress ratio of $R_{\sigma }=0$ with a maximum stress level of 350 MPa in the specimen’s net section. The tests were interrupted after a certain number of loading cycles. At each test interruption, a DIC acquisition was performed at the maximum and at the minimum applied force. After correlation, the plastic strain accumulations at the highly local deformed regions of each notch were measured. An example of the evolution of the accumulated plastic strain taken from one experimental test is reported in Figure 6. The red solid curve represents the evolution of the local maximum strain in the most deformed zones after each interruption, while the blue solid curve is the minimum strain (i.e., the residual plastic strain). The local strain features an initial stabilization after the first load reversal, followed by a rapid increment which indicates the crack nucleation and then the crack propagation. It was noted that all the final cracks nucleated in the most deformed zones highlighted by DIC analysis of the first reversal cycle.

### 3.3. Experimental Distribution of Strain Concentration Factors

**Figure 7:**
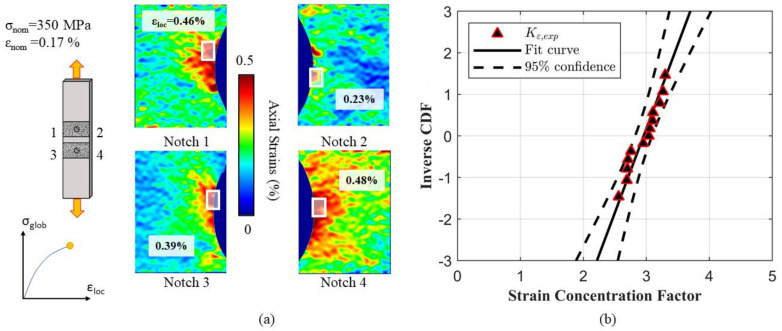
Strain heterogeneities as a function of the local microstructure. The maximum local strain was computed from DIC observation (**a**), and the obtained data were found to follow a Gaussian statistical distribution as shown in the probability plot (**b**).

As stated in the previous sections, the position of the localization of the maximum deformation is randomly distributed along the notch’s radius. This is due to the microstructure, which features randomly oriented grains that may or may not accommodate the dislocation motion. The strain maps resulting from the DIC analysis of four notches of one specimen tested at the highest applied force are reported in Figure 7. The position of the notches in relation to the local axis is schematically reported in Figure 7a. The local strains were computed as the averaged values inside the control areas as shown in Figure 7a, and the positions and values vary from one notch to another, even if the specimen is the same.

The computed strain concentration factors $K_{\varepsilon }$ were plotted into a Gaussian probability plot as shown in Figure 7b. Together with the experimental data set, Figure 7b also shows the fitted statistical distribution by a solid black line, and the mean µ and standard deviation σ parameters are reported in Table 1. As can be seen, all the experimental points fall within the 95% confidence bands plotted in dashed black lines, meaning that the chosen distribution is a good approximation of the experimental results.

## 4. Crystal Plasticity Simulations

### 4.1. Crystal Plasticity Model

Crystal Plasticity (CP) finite element simulations were performed with Warp3D [12]. This software employs an *implicit* framework to solve the global non-linear equations of nodal equilibrium, using an incremental-iterative approach. The code is based on the deformation model proposed by Asaro et al. [4,5], consisting of a multiplicative decomposition of the deformation gradient:

(2) $$F=F_{e}\cdot F_{p}$$

where $F_{e}$ and $F_{p}$ are the elastic and the plastic part of the deformation gradient, respectively. To define the current deformation state, it is necessary to define the velocity gradient, which can be broken down into its elastic and plastic parts:

(3) $$L=\dot{F}\cdot F^{-1}={\dot{F}}_{e}\cdot F_{e}{}^{-1}+F_{e}\cdot {\dot{F}}_{p}\cdot F_{p}{}^{-1}\cdot F_{e}{}^{-1}=L_{e}+L_{p}$$

According to [5], the plastic deformation of a crystal is supposed to occur by dislocation slip, hence the plastic deformation gradient $L_{p}$ can be written as:

(4) $$L_{p}=\overset{n}{\underset{\alpha =1}{\sum }}{\dot{\gamma }}^{\left(\alpha \right)}\;\cdot \;\left(s^{\left(\alpha \right)}\times m^{\left(\alpha \right)}\right)$$

where ${\dot{\gamma }}^{\left(\alpha \right)}$ is the slipping rate associated with the α-slip plane, while $s^{\left(\alpha \right)}$ and $m^{\left(\alpha \right)}$ are the unit vectors describing the slip direction and the normal to the slip plane on the α-slip system, respectively. In Warp3D, the slip rate in the α-th slip system is represented as a power law function of the resolved shear stress $\tau^{\left(\alpha \right)}$ and the slip resistance $\tilde{\tau }$:

(5) $${\dot{\gamma }}^{\left(\alpha \right)}=\frac{{\dot{\gamma }}_{0}}{\tilde{\tau }}{\left|\frac{\tau^{\left(\alpha \right)}}{\tilde{\tau }}\right|}^{n-1}\tau^{\left(\alpha \right)}$$

where ${\dot{\gamma }}_{0}$ is the reference slip rate and n is the hardening exponent (typically n is equal to 20 to force the material to approximate an independent flow rate).

Warp3D allows two different numerical methods for the computation of the slip system’s strength, which are: the phenomenological constitutive models, in which a critical resolved shear stress, $\tau_{c}{}^{\left(\alpha \right)}$, is associated with each α-slip system; and the physics-based constitutive models, in which the behaviour of the material is linked with the energy required to overcome lattice defects. In this paper, a physics-based constitutive model was used, and in particular from those available in the Warp3D routine, the Mechanical Threshold Stress model (MTS) was chosen. During the deformation, the material experiences the hardening mechanism due to the presence of obstacles that limit the mobility of the dislocations. A metal experiences four different stages of work hardening; in particular, the first hardening stage is appreciable only with material in the form of single crystals [13,14]. The MTS model provides temperature and rate dependent hardening in Stage III, while Stage IV is described by a local strain gradient of the elastic distortion. The total strength can be written in the following general form [12]:

(6) $$\tilde{\tau }=\tau_{a}+\tau_{y}\left(T,\dot{\varepsilon }\right)\;\cdot \;\frac{\mu \left(T\right)}{\mu_{0}}+\bar{\tau }\left(\varepsilon_{p},T,\dot{\varepsilon }\right)\;\cdot \;\frac{\mu \left(T\right)}{\mu_{0}}$$

where $\tau_{a}$ is the yielding stress and $\tau_{y}$ and $\bar{\tau }$ are the scale factor function of the temperature and the strain rate. The term $\bar{\tau }$ depends on the accumulated plastic strain and can be expressed as:

(7) $$\frac{d\bar{\tau }}{dt}=\theta_{0}\cdot {\left(1-\frac{\bar{\tau }}{\tau_{v}\left(T,\dot{\varepsilon \;}\right)}\right)}^{m}\overset{n}{\underset{\alpha =1}{\sum }}\left|{\dot{\gamma }}^{\left(\alpha \right)}\right|$$

where $\tau_{v}(T,\dot{\varepsilon )}$ is the saturation strength of work hardening for a given temperature and strain rate without dependence on accumulated plastic strain $\varepsilon^{p}$. The parameter $\theta_{0}$ determines the initial Stage II hardening slope. The parameters $\tau_{y}\left(T,\dot{\varepsilon }\right)$ and $\tau_{v}\left(T,\dot{\varepsilon \;}\right)$ have the following expressions:

(8) $$\tau_{y}\left(T,\dot{\varepsilon \;}\right)={\hat{\tau }}_{y}{\left[1-{\left(\frac{k\cdot T}{\mu \left(T\right)\;\cdot \;b^{3}\cdot g_{0,y}}\cdot \ln\frac{{\dot{\varepsilon }}_{0,y}}{\dot{e}}\right)}^{1/q_{y}}\right]}^{1/p_{y}}$$

(9) $$\tau_{v}\left(T,\dot{\varepsilon \;}\right)={\hat{\tau }}_{v}{\left[1-{\left(\frac{k\cdot T}{\mu \left(T\right)\;\cdot \;b^{3}\cdot g_{0,v}}\cdot \ln\frac{{\dot{\varepsilon }}_{0,v}}{\dot{e}}\right)}^{1/q_{v}}\right]}^{1/p_{v}}$$

where $\dot{e}$ has the following expression:

(10) $$\dot{e}=\sqrt{\frac{2}{3}D:D}$$

In Equations (7) and (8), *k* is the Boltzmann constant, *T* is the absolute temperature, *b* is the magnitude of the Burger’s vector, $g_{y,v}$ are the normalized activation energies, ${\dot{\varepsilon }}_{y,v}$ are the reference strain rates and $g_{y,v}$ and $p_{y,v}$ are the constants related to the shape of the activation energy barrier.

### 4.2. Crystal Plasticity Model

The CP model’s parameters were fitted from the tensile curves presented in Section 3.1. The strategy adopted consists of a trial and error procedure, in which the tensile local curve of the ROI obtained from DIC was compared with that obtained from FE simulation.

**Figure 8:**
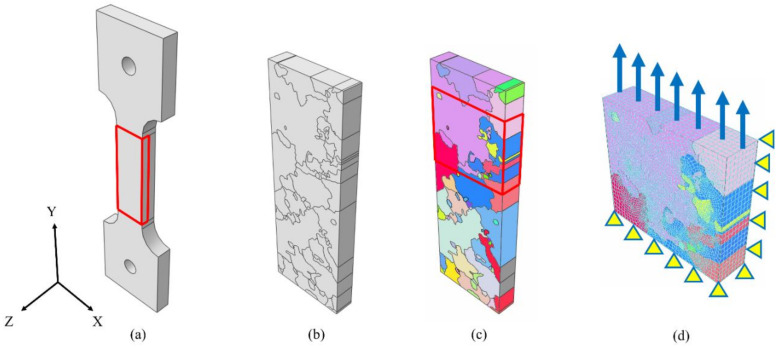
CP simulations of tensile specimens’ scheme: (**a**) whole specimen geometry with the half parallel section numerically simulated highlighted in red; (**b**) model of the half parallel section simulated considering the microstructure; (**c**) EBSD representation of the simulated model; (**d**) reduced model aimed to simulate the ROI investigated experimentally via DIC.

To obtain the numerical tensile curves, a precise simulation of the specimen’s microstructure was required. As highlighted in Section 2.2, a grain observed on a certain face could result in a totally different shape or even disappear on the second face, resulting in a 3D texture. A simulation in which the microstructure is fully reconstructed even in the thickness direction could be prohibitive from the point of view of both numerical effort and the difficulty of obtaining an experimental 3D map of the grains. To overcome these limitations, the microstructure was assumed to remain the same as that observed on the flat surface until the mid-plane of the specimen (i.e., columnar grains). An example of the procedure adopted to reconstruct one half of the parallel section of the tensile specimen is reported in Figure 8a,b.

To reduce the computational effort, only the part of the “half-parallel-section” which corresponds to the observed ROI was numerically simulated with the DIC in in-situ configuration. An example of one simulated ROI is provided in Figure 8c. The surface microstructure was obtained by optical observations after chemical etching. This was performed in order to avoid possible distortions of the grains due to EBSD analysis, which is particularly evident when scanning large areas. From the microscope images, the grains map was mapped manually. Then, using a Matlab code, the grains map was converted into a cloud of points, which was then imported into Abaqus/CAE 6.14 via a Python script. The points were then connected in order to form closed curves, which were then used to create partitions that can be individually meshed. The models were meshed with the dedicated module available in Abaqus/CAE 6.14, and one of the meshed models obtained is shown in Figure 8d. The models were meshed with linear hexahedral FEs, with a mean element size of 0.15 mm.

The ROI models were simulated in Warp3D [12] by considering the following loading and boundary conditions:

- Fixed displacement along the Y direction for all the nodes lying on the XZ plane.
- Fixed displacement along the Z direction for all the nodes lying on the XY plane.
- Fixed displacement along the X direction for the nodes positioned at the origin to avoid numerical errors.
- An imposed displacement along the Y direction for all the nodes that belong to the upper model surface.

**Figure 9:**
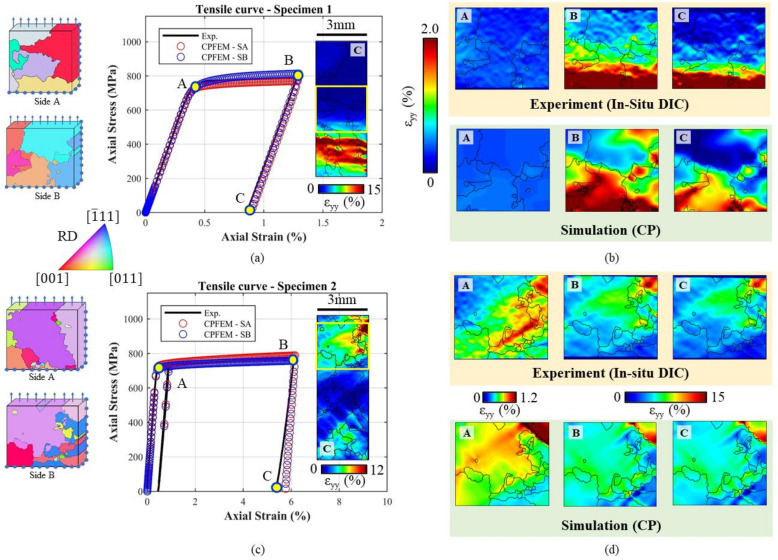
Comparison between CP and finite element (FE) simulations of the tensile specimens and the experimental results obtained: (**a**) macroscopic tensile behaviour of the first tensile specimen with the ex-situ DIC strain map; (**b**) comparison between the microscopic results at specific locations on the tensile curve from FE and experimental testing of the first specimen; (**c**) macroscopic tensile behaviour of the second tensile specimen with the ex-situ DIC strain map; (**d**) comparison between the microscopic results at specific locations on the tensile curve from FE and experimental testing of the second specimen.

The experimental tensile curves were obtained considering the average strain computed by DIC analysis, that is hence representative of the observed region taking into account the specimen’s whole thickness. To take into account the effect of the microstructure on the specimen’s thickness, the back face was also simulated using CP. The specimen’s Side A was meshed with 4962 nodes and 3925 elements, while Side B was meshed with 15,936 nodes and 13,070 elements. The two numerical curves obtained were compared with the experimental one, and the CP parameters were changed until satisfactory fitting was obtained. The final curves obtained in the last iteration were plotted against the experimental ones in Figure 9a, where Side B corresponds to the specimen’s face observed using DIC. The macroscopic tensile behaviour of the material is well reproduced by the FE simulation, featuring a maximum error of 3.29%. The microscopic behaviour is reported in Figure 9b for three relevant points, A, B and C, by comparing the experimental and the numerical strain maps. The strain maps simulated using FE are also satisfactory in relation to the experimental findings for both the points considered. The parameters of the model obtained are reported in Table 2.

The second tensile specimen was simulated using CP before testing, employing the material parameters found in the first test. This makes it possible to have a general idea of the local strain accumulation. The analysis showed that the big upper grain on Side B was expected to undergo high local deformation, and for this reason, the ROI by DIC was positioned in this region. The specimen’s Side A was meshed with 33,759 nodes and 29,536 elements, while Side B was meshed with 41,650 nodes and 36,819 elements. The results of the DIC in an in-situ configuration of the selected region confirmed the FE estimations. The results of the macroscopic tensile curves of the simulated ROIs for the two faces are compared with the experimental curve in Figure 9c; the comparison between the experimental and numerical strain maps is then shown in Figure 9d. From both the macroscopic and microscopic point of view, the FE simulations of the second tensile specimen agree well with the experimental findings.

### 4.3. Simulation of the Notched Specimens

The variability of the strain concentration factor (SCF) obtained from the experimental results in Section 3.3 has to be attributed to the random microstructure. To reproduce the experimental scatter, a series of FE simulations is required, taking into account the variability due to the microstructure. The notched specimens tested were then simulated using the CP model fitted in Section 4.1.

**Figure 10:**
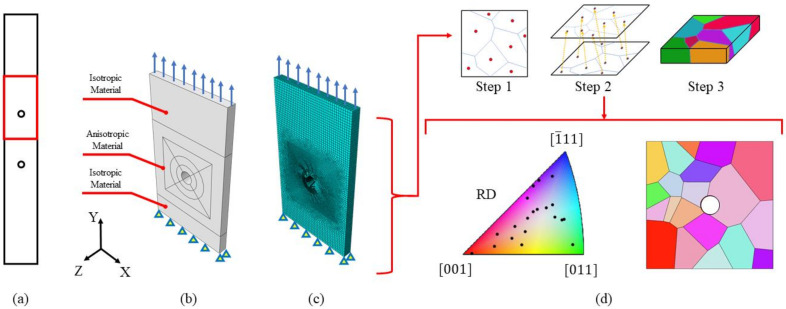
Scheme of the simulated model to reproduce the experimental material scatter: (**a**) whole scheme of the specimen considered with the simulated geometry highlighted in red; (**b**) adopted numerical models to describe the behaviour of sections of the specimen; (**c**) final meshed model with boundary and loading conditions. The model was meshed with linear hexahedron FEs, resulting in 117,887 nodes and 104,960 elements; (**d**) EBSD of the obtained random microstructure with the orientation of the grains inside the stereographic triangle.

To reduce the numerical efforts, only one half of the specimen was simulated. To further simplify the model, only the portion of material surrounding the notch was modelled in terms of the microstructure, while the remaining material was considered to have isotropic behaviour. The schematic of the specimen considered and the different material models adopted in the simulations are reported in Figure 10a,b respectively. The position and shape of each grain were obtained by using the Voronoi tessellation method, which has been shown to reproduce the microstructure of metallic materials well [18,19,20]. This methodology consists of dividing the space into sub-regions, which represent the locus of the points with the minimum distance from the one specific seed. The random aggregation of grains was obtained using a Matlab code. This code makes it possible to obtain a microstructure that features: (i) random grain orientation and position of the corresponding seeds, and (ii) random growing direction of the grains. A random growing direction of the grains was chosen to approximate the real grain geometry in the thickness direction.

The algorithm starts by randomly distributing the grains’ seeds. This was obtained by drawing a number of coordinates from a uniform random distribution, limited to the feasible domain represented by the specimen’s external surfaces. A growing direction was associated with each seed by randomly drawing two angles from a uniform distribution, limited between −20° and 20°. The number of drawings was calculated as the average grain density times the surface area considered. The grain orientations were assigned by changing the value of the Euler’s angles in a random way by extractions from uniform distributions obtained from the values of the EBSD analyses. The first layer of FE elements was associated with belonging to a certain grain, according to the partitions obtained from the Voronoi tessellation of the surface. Once the first layer of FE elements was associated with a certain grain index, the analysis continued until the last layer. The positions of the initial seeds were updated for each layer according to the growing direction. The three main steps performed by the developed algorithm are schematically reported in Figure 10d, where the finite elements, shown in Figure 10c, were mapped into a random microstructure. The final microstructure features coarse boundaries, represented by the elements’ faces. The orientations of the grains for the obtained EBSD map are reported in black dots inside the stereographic triangle in Figure 10d, and as it can be noted, they are well distributed inside the domain. In reality, the grains are smoother, but it was demonstrated that the approximation of considering the boundary surfaces to be rough did not influence the results [20].

Once the geometry and the microstructure of the specimens were randomly generated, a total of ten CP simulations were run using the free finite elements code Warp3D [12]. From each simulation, a set of four strain concentration factors can be computed along the edge of the notch: two from the frontal face and two from the rear face. Each simulation was obtained by imposing a nominal stress of 350 MPa on the top face in order to reproduce the experimental conditions described in Section 3.3. To be consistent with the experimental findings presented in Section 3.3, the numerical values of the strain concentration factors were computed by averaging the FE nodes inside a control area of 210 × 310 ${\mu m}^{2}$. Due to the relatively large number of notched surfaces, the computation of the $K_{\varepsilon }$s was performed using Matlab scripts that are able to automatically locate the most critical surface control area. The script considers a moving window that scans all the nodes inside a circular crown with a thickness of 0.21 mm. The lower and the upper bounds of the windows have a relative angle, which is computed in order to make the area of the moving window the same as that used in the experimental characterization of $K_{\varepsilon }$s from DIC analyses. At each iteration, the nodal stresses inside the window were averaged; the location of the window with the highest averaged stress was considered to be critical, and hence it was the one for which the strain intensity factor was to be evaluated.

**Figure 11:**
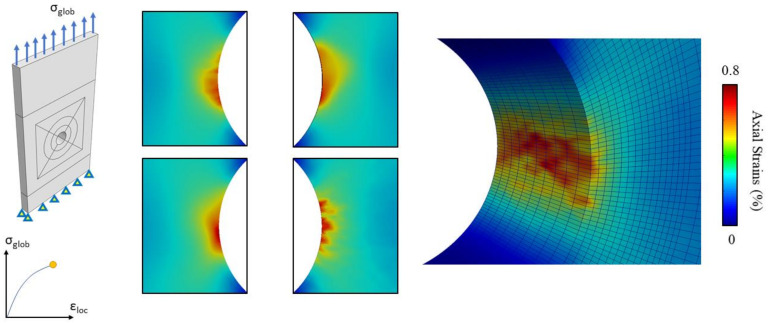
Results of CPFE simulation of the notched specimen. The axial strain concentrations observed experimentally via DIC analysis are well estimated by the numerical simulations, considering the effect of random microstructure generated using a Matlab code.

In Figure 11, the results in terms of total axial strain of one of the ten FE simulations are reported. Comparing these results with the strain maps obtained via DIC analysis, it is possible to note how the different strain concentrations are well reproduced via numerical simulations.

## 5. Discussion

**Figure 12:**
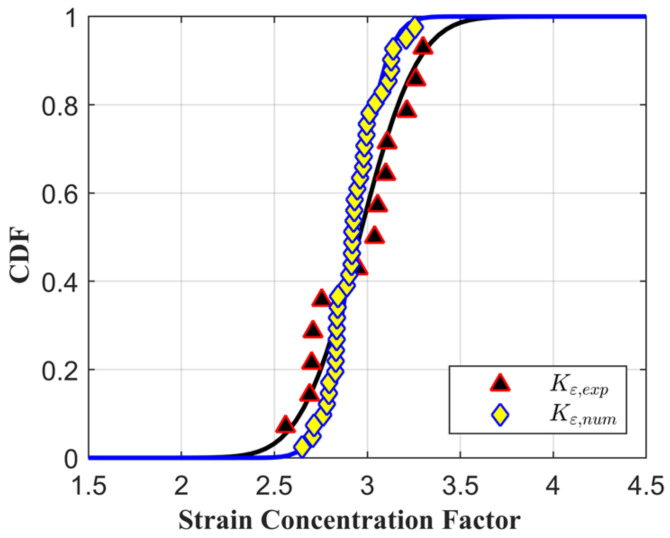
Comparison between the distribution of the strain concentration factor estimated experimentally using DIC and that calculated numerically using CPFE.

The strain concentration factors $K_{\varepsilon }$ computed from the numerical simulations of the notched specimens are compared with the experimental values in Figure 12, and the comparison is provided in terms of cumulative density function. The fitted distributions are shown by a solid black line and a solid blue line for the experimental and the numerical data, respectively.

The fitted statistical distribution is a Gaussian distribution, and the slopes of the simulated and numerical distributions are very similar. The fitted parameters of the experimental data set and the numerical ones are reported in Table 3; the mean value of the two distributions is very similar, while the experimental standard deviation is almost double the numerical one. From the numerical CP simulations, the local tensile curves for the most stressed region of the notches are available; knowing the maximum level of stress and strain and the value of the stress and strain concentration factors, the local hysteresis cycle can be estimated using Neuber’s rule supposing an elastic shake down. The local stress and strain concentration factors will be:

(11) $$\begin{array}{l} K_{\sigma }=\frac{\sigma_{FEM,loc}}{\sigma_{n}},\;\\ K_{\varepsilon }=\frac{\varepsilon_{FEM,loc}}{\varepsilon_{n}} \end{array}$$

where $\sigma_{FEM,loc}$ and $\varepsilon_{FEM,loc}$, respectively, are the local stress and strain in the most stressed region of the notch computed from the FE simulation, and $\sigma_{n}$ and $\varepsilon_{n}$, respectively, are the remote stress and strain applied to the specimen. The stress and strain range can be computed simply using Equation (12):

(12) $$\left\{\begin{array}{c} \Delta \sigma =\sqrt{K_{\sigma }\cdot K_{\varepsilon }\cdot {\left(\Delta \sigma_{n}\right)}^{2}}\\ \Delta \varepsilon =\Delta \sigma /E \end{array}\right.$$

Knowing the stress and strain ranges of each numerical simulation, the number of cycles to failure can be computed. In this work, the Fatemi–Socie (FS) multiaxial criterion was adopted. This criterion was chosen due to the non-zero mean stress level of the hysteresis loops. The parameters of the Coffin–Manson curve adopted for estimating the fatigue lives are not reported due to intellectual property rights. The experimental results of the fatigue lives of the notched specimens are reported in Table 4; the number of cycles to failure corresponds to that after which a fatigue crack can be visible, detected using DIC analysis.

The fatigue data reported in Table 4 can be fitted using a log-normal distribution. The standard deviation of the log-life is shown to be $\sigma^{exp}{}_{logN}=0.3623$. The dispersion of the fatigue life of the specimens tested can be divided into two main contributions: (i) the first is due to the coarse microstructure that induced a variability of strain accumulation which is accentuated by the notch; (ii) the second contribution is due to the intrinsic fatigue scatter of the material which comes from tests of a uniformly stressed material. This observation can be expressed in mathematical terms as:

(13) $$\sigma^{exp}{}_{logN}=\sqrt{\sigma_{logN,K\varepsilon }{}^{2}+\sigma_{logN,mat}{}^{2}}$$

where $\sigma_{logN,K\varepsilon }$ is the fatigue scatter due to the combined effect of the microstructure and the notch effect, and $\sigma_{logN,mat}$ is the intrinsic scatter of the material. From the fatigue life estimations considering the hysteresis cycles approximated using Neuber’s rule and the variability of the $K_{\varepsilon }$, the fatigue scatter is found to be $\sigma_{logN,K\varepsilon }=0.1809$. Considering the expression in Equation (13) and the experimental data for the notched samples, the fatigue scatter associated with the material will be $\sigma_{logN,mat}=0.3139$. This value is in line with the fatigue scatter from Low Cycle Fatigue (LCF) data of smooth specimens. For a typical engineering material, such as 9CrMo steel, for which data are analysed by Beretta et al. in [21] and by Zhu et al. in [22], the scatter on the log life is about 0.1325; this value is much lower than the one found for René80, meaning that a design based only on the Coffin–Manson curve is not enough for critical fatigue components.

In light of the previous observations, during the design phase of a critical mechanical component featuring one or more notches (such as the air ducts of a gas turbine blade, which are of the same order of dimension as the considered microstructure), one has to take into account both the variability of fatigue life of the uniformly stressed material and the scatter of the stress and strain accumulation due to the interaction of the microstructure and geometrical features.

## 6. Conclusions

In this work, the statistical variability of the strain concentration factor in notched specimens of René80 superalloy is analysed and discussed. This superalloy features a coarse-grained microstructure that is mainly responsible for the high scatter in both strain localizations and fatigue life. To correctly model the interaction of the notches with the microstructure, a Crystal Plasticity model was fitted with the tensile testing of micro specimens. The local microstructural deformation was evaluated using DIC, and the strain maps obtained were compared with numerical simulations, showing a good correlation. The model was then used to estimate the scatter of the strain concentration due to the interaction between the notches and the microstructure. From these results, it was then possible to estimate the fatigue scatter associated with the notch effect. The following conclusions hold:

- Crystal Plasticity finite element simulations showed high accuracy in the estimation of strain concentrations inside the material’s microstructure.
- The strain maps computed using CP showed good accuracy in relation to the experimental ones evaluated using the DIC technique.
- The numerical estimations of the strain concentration factors from finite element simulations of the notched specimens considering random microstructures can be well described by means of a Gaussian distribution. The numerical distribution is in accordance with the experimental distribution computed from DIC acquisitions.
- In the design phase of a critical component which features a coarse-grained microstructure of the same dimensional order of geometrical features, such as René80, the failure probability has to be computed, taking into account not only the fatigue scatter distribution of the flat specimens, but also the material scatter due to the interaction between the notches and the microstructure.

## Figures and Tables

**Table 1:** Parameters of the Gaussian statistical distribution describing the experimental strain concentration factors $K_{\varepsilon }$.

*µ*	*σ*
2.96	0.25

**Table 2:** Fitted parameters of the CP model for describing the mechanical behaviour of René80.

Property	Description	Type	Value
E	Young’s modulus	Fitted	185.0 MPa
ν	Poisson’s ratio	Fitted	0.33
$\mu_{0}$	Shear modulus	Fitted	75.5 MPa
b	Burgers’ vector	Literature [15]	3.5 × 10^−7^ mm
${\hat{\tau }}_{a}$	Athermal slip resistance	Literature [12]	0 MPa
${\hat{\tau }}_{y}$	MTS strength for intrinsic barrier (yield)	Fitted	330.0 MPa
$g_{0,y}$	Normalized activation energy for intrinsic barriers	Literature [15]	0.37
$q_{y}$	Shape coefficient for intrinsic barriers	Literature [16]	1.5
$p_{y}$	Shape coefficient for intrinsic barriers	Literature [16]	1.5
$\varepsilon_{0,y}$	Strain rate sensitivity for intrinsic barriers	Literature [17]	1.0 × 10^−9^ s^−1^
${\hat{\tau }}_{\nu }$	MTS strength for work hardening	Fitted	13.0 MPa
$g_{0,\nu }$	Normalized activation energy for work hardening	Literature [15]	1.6
$q_{\nu }$	Shape coefficient for work hardening	Literature [16]	0.667
$p_{\nu }$	Shape coefficient for work hardening	Literature [16]	1.2
$\varepsilon_{0,\nu }$	Strain rate sensitivity for work hardening	Literature [17]	1 × 10^−7^ s^−1^
$\vartheta_{0}$	Initial hardening slope	Fitted	60.0 MPa
$k_{0}$	Geometric hardening parameter	Fitted	1.0

**Table 3:** Parameters of the Gaussian statistical distribution describing the experimental and numerical strain concentration factors $K_{\varepsilon }$.

	*µ*	*σ*
**Experimental**	2.96	0.25
**Numerical**	2.93	0.14

**Table 4:** Number of cycles required to detect a visible crack using DIC analysis. All the specimens were tested with a nominal alternate stress of 350 MPa and a stress ratio Rσ = 0.

Notch Index	Crack	Cycles
R1_N1	Yes	50,000
R1_N2	Yes	50,000
R1_N3	Yes	50,000
R1_N4	Yes	50,000
R2_N1	Yes	20,000
R2_N2	Yes	5000
R2_N3	Yes	30,000
R2_N4	Yes	30,000
R3_N1	Yes	10,000
R3_N2	No	NA
R3_N3	No	NA
R3_N4	No	NA
R4_N1	No	NA
R4_N2	No	NA
R4_N3	Yes	10,000
R4_N4	Yes	10,000

## Data Availability

The data presented in this study are available on request from the corresponding author. The data are not publicly due to disclosure agreement.

## Nomenclature

${\hat{\tau }}_{a}$	Athermal slip resistance
${\hat{\tau }}_{y}$	MTS strength for intrinsic barrier (yield)
${\hat{\tau }}_{\nu }$	MTS strength for work hardening
$K_{\varepsilon }$	Strain concentration factor
$\varepsilon_{\mathrm{glob}}$	Global applied strain
$\varepsilon_{\mathrm{loc}}$	Local strain
$g_{0,y}$	Normalized activation energy for intrinsic barriers
$g_{0,\nu }$	Normalized activation energy for work hardening
$k_{0}$	Geometric hardening parameter
$p_{y}$	Shape coefficient for intrinsic barriers
$p_{\nu }$	Shape coefficient for work hardening
$q_{y}$	Shape coefficient for intrinsic barriers
$q_{\nu }$	Shape coefficient for work hardening
$\varepsilon_{0,y}$	Strain rate sensitivity for intrinsic barriers
$\varepsilon_{0,\nu }$	Strain rate sensitivity for work hardening
$\mu_{0}$	Shear modulus
$\vartheta_{0}$	Initial hardening slope
µ	Mean of the Gaussian statistical distribution
b	Burgers’ vector
E	Young’s modulus
ν	Poisson’s ratio
σ	Standard deviation of the Gaussian statistical distribution

## Acronyms

CP	Crystal plasticity
FE	Finite element
SCF	Strain concentration factor

## References

[1] Antolovich B.F. (2018). Fatigue and Fracture of Nickel-Base Superalloys. Fatigue Fract..

[2] Gottschalk H., Schmitz S., Seibel T., Rollmann G., Krause R., Beck T. (2015). Probabilistic Schmid factors and scatter of low cycle fatigue (LCF) life. Materwiss. Werksttech..

[3] Engel B., Mäde L., Lion P., Moch N., Gottschalk H., Beck T. (2019). Probabilistic modeling of slip system-based shear stresses and fatigue behavior of coarse-grained ni-base superalloy considering local grain anisotropy and grain orientation. Metals.

[4] Asaro R.J. (1983). Crystal plasticity. J. Appl. Mech..

[5] Asaro R.J. (1983). Micromechanics of Crystals and Polycrystals. Adv. Appl. Mech..

[6] Dunne F.P.E., Wilkinson A.J., Allen R. (2007). Experimental and computational studies of low cycle fatigue crack nucleation in a polycrystal. Int. J. Plast..

[7] Lim H., Carroll J.D., Battaile C.C., Buchheit T.E., Boyce B.L., Weinberger C.R. (2014). Grain-scale experimental validation of crystal plasticity finite element simulations of tantalum oligocrystals. Int. J. Plast..

[8] Jiang J., Dunne F.P.E., Britton T.B. (2017). Toward Predictive Understanding of Fatigue Crack Nucleation in Ni-Based Superalloys. JOM.

[9] Delaire F., Raphanel J.L., Rey C. (2000). Plastic heterogeneities of a copper multicrystal deformed in uniaxial tension: Experimental study and finite element simulations. Acta Mater..

[10] Battaile C.C., Emery J.M., Brewer L.N., Boyce B.L. (2015). Crystal plasticity simulations of microstructure-induced uncertainty in strain concentration near voids in brass. Philos. Mag..

[11] Prithivirajan V., Sangid M.D. (2018). The role of defects and critical pore size analysis in the fatigue response of additively manufactured IN718 via crystal plasticity. Mater. Des..

[12] Healy B. WARP3D. http://www.warp3d.net/.

[13] Rollett A.D., Kocks U.F. (1993). A Review of the Stages of Work Hardening. Solid State Phenom..

[14] Mecking H., Kocks U.F. (1981). Kinetics of flow and strain-hardening. Acta Metall..

[15] Gray G.T., Chen S.R., Vecchio K.S. (1999). Influence of grain size on the constitutive response and substructure evolution of MONEL 400. Metall. Mater. Trans. A.

[16] Kok S., Beaudoin A.J., Tortorelli D.A. (2002). A polycrystal plasticity model based on the mechanical threshold. Int. J. Plast..

[17] Banerjee B. (2007). The Mechanical Threshold Stress model for various tempers of AISI 4340 steel. Int. J. Solids Struct..

[18] Zhang P., Balint D., Lin J. (2011). An integrated scheme for crystal plasticity analysis: Virtual grain structure generation. Comput. Mater. Sci..

[19] Zhang P., Karimpour M., Balint D., Lin J., Farrugia D. (2012). A controlled Poisson Voronoi tessellation for grain and cohesive boundary generation applied to crystal plasticity analysis. Comput. Mater. Sci..

[20] Zhang K.S., Ju J.W., Li Z., Bai Y.L., Brocks W. (2015). Micromechanics based fatigue life prediction of a polycrystalline metal applying crystal plasticity. Mech. Mater..

[21] Beretta S., Foletti S., Rusconi E., Riva A., Socie D. (2016). A log-normal format for failure probability under LCF: Concept, validation and definition of design curve. Int. J. Fatigue.

[22] Zhu S.P., Foletti S., Beretta S. (2017). Probabilistic framework for multiaxial LCF assessment under material variability. Int. J. Fatigue.

